# Supplementary material for: Cortex cis-regulatory switches establish scale colour identity and pattern diversity in Heliconius
Source: eLife. 2021 Jul 19;10:e68549. doi: 10.7554/eLife.68549 (PMC8289415; doi:10.7554/eLife.68549)
Supplement: Figure 2—source data 1. — Both SNPs were contained in the protein-coding sequence of the gene Cortex. Individuals from the RNA-seq experiment match the genotype of the source populations. [file elife-68549-fig2-data1.docx]

| Sequence | Race | Individual | Informative site, scaffold 215006 | |
| --- | --- | --- | --- | --- |
|  |  |  | 1207068 | 1210502 |
| WGS | *melpomene* | P3_1 | TT | TT |
|  |  | P3_2 | TT | TT |
|  |  | P3_3 | TC | TC |
| WGS | *rosina* | ros10_1 | CC | CC |
|  |  | ros10_2 | CC | CC |
|  |  | ros10_3 | CC | CC |
|  |  | ros10_4 | CC | CC |
|  |  | ros10_5 | CC | CC |
|  |  | ros10_6 | CC | CC |
|  |  | ros10_7 | CC | CC |
|  |  | ros10_8 | CC | CC |
|  |  | ros10_9 | CC | CC |
|  |  | ros10_10 | CC | CC |
| RNAseq | *melpomene* | 49 | TT | TT |
|  |  | 52 | TT | TT |
|  |  | 54 | TT | TT |
|  |  | 55 | TT | TT |
|  |  | 56 | TT | TT |
|  |  | 61 | TT | TT |
|  |  | 62 | TT | TT |
| RNAseq | *rosina* | 13 | CC | CC |
|  |  | 14 | CC | CC |
|  |  | 15 | CC | CC |
|  |  | 16 | CC | CC |
|  |  | 17 | CC | CC |
|  |  | 24 | CC | CC |
