# Supplementary material for: Cortex cis-regulatory switches establish scale colour identity and pattern diversity in Heliconius
Source: eLife. 2021 Jul 19;10:e68549. doi: 10.7554/eLife.68549 (PMC8289415; doi:10.7554/eLife.68549)
Supplement: Figure 2—source data 2. — Once again, all individuals match the expected genotype. [file elife-68549-fig2-data2.docx]

|  |  | Informative site, scaffold 1505 | |
| --- | --- | --- | --- |
| Sequence | Race | Individual | 2306177 |
| WGS | *hydara* | STRI_WOM_0039 | AA |
|  |  | STRI_WOM_0040 | AA |
|  |  | STRI_WOM_0042 | AA |
|  |  | STRI_WOM_0088 | AA |
|  |  | STRI_WOM_5193 | AA |
|  |  | STRI_WOM_5351 | AA |
| WGS | *demophoon* | Pet_ED3 | GG |
|  |  | Pet_ED4 | GG |
|  |  | Pet_ED5 | GG |
|  |  | Pet_ED6 | GG |
|  |  | STRI_WOM_0033 | GG |
|  |  | STRI_WOM_0082 | GG |
|  |  | STRI_WOM_0087 | GG |
|  |  | STRIWOM1284 | GG |
|  |  | STRIWOM5353 | GG |
|  |  | STRIWOM5362 | GG |
| RNAseq | *hydara* | 17 | AA |
|  |  | 25 | AA |
|  |  | 33 | AA |
|  |  | 34 | AA |
|  |  | 36 | AA |
| RNAseq | *demophoon* | A4 | GG |
|  |  | D2 | GG |
|  |  | D6 | GG |
|  |  | D9 | GG |
|  |  | C6 | GG |
|  |  | H3 | GG |
|  |  | A4 | GG |
