# Supplementary material for: Cortex cis-regulatory switches establish scale colour identity and pattern diversity in Heliconius
Source: eLife. 2021 Jul 19;10:e68549. doi: 10.7554/eLife.68549 (PMC8289415; doi:10.7554/eLife.68549)
Supplement: Figure 2—source data 3. [file elife-68549-fig2-data3.docx]

| **Locus** | **PCR Primers (5’ > 3’)** |
| --- | --- |
| ***eF1***$\boldsymbol{\alpha}$ | AAGAATTCCCTCCCCTCGGT  CCACCAGCACCTTCCTTGAA |
| ***rpL3*** | AAGTCCCTTCGTGTCCACAC  GTGTCTGGAAGCGACCATGT |
| ***polyABP*** | CTACGCACATCTCTCGGAGC  CAGCTGGACGTCCACTTGTA |
| ***cortex*** | ATAGGACTGGCGAGCTGGTA  TTCGTACTCGGTCCAGTCCA |
