# Supplementary material for: Cortex cis-regulatory switches establish scale colour identity and pattern diversity in Heliconius
Source: eLife. 2021 Jul 19;10:e68549. doi: 10.7554/eLife.68549 (PMC8289415; doi:10.7554/eLife.68549)
Supplement: Figure 2—source data 4. [file elife-68549-fig2-data4.docx]

| **Hera1 Gene ID** | **gene name / description** | **Hmel2 Gene ID** |
| --- | --- | --- |
| evm.model.Herato1505.61 | Acylpeptide | HMEL000003 |
| evm.model.Herato1505.62 | Trehalase-1B | HMEL000004 |
| evm.model.Herato1505.63 | Trehalase-1A  B9_protein | HMEL000006 |
| evm.model.Herato1505.64 | B9_protein | HMEL000007 |
| evm.model.Herato1505.65 | HM0008_nov | HMEL000008 |
| evm.model.Herato1505.66 | WD40_repeat_d_85 | HMEL000010 |
| evm.model.Herato1505.67 |  |  |
| evm.model.Herato1505.68 | CG2519 | HMEL000012  HMEL000012 |
| evm.model.Herato1505.69 | Unkempt | HMEL000013 |
| evm.model.Herato1505.70 | Histone_H3 | HMEL000014 |
| evm.model.Herato1505.71 |  |  |
| evm.model.Herato1505.72 | HM00015_nov | HMEL000015 |
| evm.model.Herato1505.73 | HM00016_nov | HMEL000016 |
| evm.model.Herato1505.74 | RecQ_helicase | HMEL000017 |
| evm.model.Herato1505.75 |  |  |
| evm.model.Herato1505.76 | HM00018_nov | HMEL000018 |
| *****(1505.35!)***** | BmSuc2 | HMEL000019 |
| evm.model.Herato1505.77 | CG5796 | HMEL000020 |
| evm.model.Herato1505.78 | HM00021_nov | HMEL000021 |
| evm.model.Herato1505.79 | Enoyl-coA-hydratase | HMEL000022 |
| evm.model.Herato1505.80 |  |  |
| evm.model.Herato1505.81 | ATP-bp  ****** | HMEL002023 |
| evm.model.Herato1505.82 |  |  |
| evm.model.Herato1505.83 | HMEL002023 |  |
| evm.model.Herato1505.84 | HM00024_nov | HM00024_nov |
|  |  | g8186 |
| evm.model.Herato1505.85 | Cortex | HMEL000025 |
| evm.model.Herato1505.86 | parn | HMEL000026 |
| evm.model.Herato1505.87 | CG31320 | HMEL000027 |
| evm.model.Herato1505.88 | Manf | HMEL000028 |
| evm.model.Herato1505.89 | CG4692 | HMEL000029 |
| evm.model.Herato1505.90 | Psmd4 | HMEL000030 |
|  | HM000031_nov | HMEL000031 |
| evm.model.Herato1505.91  evm.model.Herato1505.90 | Rnz | HMEL000032 |
| evm.model.Herato1505.92 | Lmtk1 | HMEL000033 |
| evm.model.Herato1505.93 | Wdr13 | g8202 |
| evm.model.Herato1505.94 | Dome1 | HMEL013472 |
| evm.model.Herato1505.95 | Washout | HMEL000036 |
| evm.model.Herato1505.96 | Dome2 | HMEL000037 |
| evm.model.Herato1505.97 | lethal_2_k05819 | g8206 |
| evm.model.Herato1505.98 | MKK3 | HMEL000039 |
| evm.model.Herato1505.99 | ERCC-6 | HMEL000040 |
| evm.model.Herato1505.100 | Penguin | HMEL000041 |
| evm.model.Herato1505.101 | Thymidilate_kinase | HMEL000042 |
| evm.model.Herato1505.102 | Cas_activated_DNAse | HMEL000043 |
| evm.model.Herato1505.103 | Ribosome_biosynth_r | HMEL000044 |
| evm.model.Herato1505.104 | CG12659 | HMEL000045 |
| evm.model.Herato1505.105 | CG33505 | HMEL000046 |
| evm.model.Herato1505.106 | Sr | HMEL000047 |
| evm.model.Herato1505.107 | Hm00048_nov | HMEL000048 |
| evm.model.Herato1505.108 | Hm00049_nov | HMEL000049 |
| evm.model.Herato1505.109 |  | HMEL000050 |
|  | Papillin | HMEL000051 |
| evm.model.Herato1505.110 | HM000052_nov | HMEL000052 |
