# Supplementary material for: Cortex cis-regulatory switches establish scale colour identity and pattern diversity in Heliconius
Source: eLife. 2021 Jul 19;10:e68549. doi: 10.7554/eLife.68549 (PMC8289415; doi:10.7554/eLife.68549)
Supplement: Figure 7—source data 1. [file elife-68549-fig7-data1.docx]

| Accession | Country | Genus | Species | Subsp |
| --- | --- | --- | --- | --- |
| ERS5932155 | Panama | Heliconius | erato | demophoon |
| ERS5932158 | Panama | Heliconius | erato | hydara |
| ERS5932159 | Panama | Heliconius | erato | hydara |
| ERS5932160 | Panama | Heliconius | erato | hydara |
| ERS5932156 | Panama | Heliconius | erato | demophoon |
| ERS5932164 | Panama | Heliconius | melpomene | rosina |
| ERS5932158 | Panama | Heliconius | melpomene | melpomene |
| ERS5932165 | Panama | Heliconius | melpomene | rosina |
| ERS5932162 | Panama | Heliconius | melpomene | melpomene |
| ERS5932157 | Panama | Heliconius | erato | demophoon |
| ERS5932163 | Panama | Heliconius | melpomene | melpomene |
| ERS5932166 | Panama | Heliconius | melpomene | rosina |
