# Supplementary material for: Cortex cis-regulatory switches establish scale colour identity and pattern diversity in Heliconius
Source: eLife. 2021 Jul 19;10:e68549. doi: 10.7554/eLife.68549 (PMC8289415; doi:10.7554/eLife.68549)
Supplement: Figure 8—source data 1. [file elife-68549-fig8-data1.docx]

| **Accession** | **Country** | **Genus** | **Species** | **Subsp** |
| --- | --- | --- | --- | --- |
| ERS4212889 | Colombia | Heliconius | melpomene | bellula |
| ERS4212890 | Colombia | Heliconius | melpomene | bellula |
| ERS4212589 | Colombia | Heliconius | melpomene | bellula |
| ERS4212891 | Colombia | Heliconius | melpomene | bellula |
| ERS4212892 | Colombia | Heliconius | melpomene | bellula |
| ERS2196350 | Colombia | Heliconius | melpomene | malleti |
| ERS1030547 | Colombia | Heliconius | melpomene | malleti |
| ERS1030548 | Colombia | Heliconius | melpomene | malleti |
| ERS2196351 | Colombia | Heliconius | melpomene | malleti |
| ERS1030546 | Colombia | Heliconius | melpomene | malleti |
| ERS4212641 | Colombia | Heliconius | timareta | tristero |
| ERS4212642 | Colombia | Heliconius | timareta | tristero |
| ERS4212643 | Colombia | Heliconius | timareta | tristero |
| ERS4212644 | Colombia | Heliconius | timareta | tristero |
| ERR2298254 | Peru | Heliconius | melpomene | amaryllis |
| ERR2298255 | Peru | Heliconius | melpomene | amaryllis |
| ERR2298256 | Peru | Heliconius | melpomene | amaryllis |
| ERR2298257 | Peru | Heliconius | melpomene | amaryllis |
| ERR2298260 | Peru | Heliconius | melpomene | amaryllis |
| ERR2298261 | Peru | Heliconius | melpomene | amaryllis |
| ERR2298205 | Panama | Heliconius | melpomene | rosina |
| ERR2298206 | Panama | Heliconius | melpomene | rosina |
| ERR2298207 | Panama | Heliconius | melpomene | rosina |
| ERR2298208 | Panama | Heliconius | melpomene | rosina |
| ERR2298209 | Panama | Heliconius | melpomene | rosina |
| ERR2298210 | Panama | Heliconius | melpomene | rosina |
| ERR2298211 | Panama | Heliconius | melpomene | rosina |
| ERR2298219 | Panama | Heliconius | melpomene | rosina |
| ERR2298221 | Panama | Heliconius | melpomene | rosina |
| ERR2298262 | Peru | Heliconius | timareta | thelxinoe |
| ERR2298263 | Peru | Heliconius | timareta | thelxinoe |
| ERR2298264 | Peru | Heliconius | timareta | thelxinoe |
| ERR2298265 | Peru | Heliconius | timareta | thelxinoe |
| ERR2298266 | Peru | Heliconius | timareta | thelxinoe |
| ERS4212893 | Colombia | Heliconius | erato | dignus |
| ERS4212895 | Colombia | Heliconius | erato | dignus |
| ERS4212896 | Colombia | Heliconius | erato | dignus |
| ERS4212581 | Colombia | Heliconius | erato | dignus |
| ERS4212897 | Colombia | Heliconius | erato | dignus |
| SRR4031993 | Colombia | Heliconius | erato | chestertonii |
| SRR4032020 | Colombia | Heliconius | erato | chestertonii |
| SRR4032031 | Colombia | Heliconius | erato | chestertonii |
| SRR4032104 | Colombia | Heliconius | erato | chestertonii |
| SRR4032105 | Colombia | Heliconius | erato | chestertonii |
| SRR4031996 | Colombia | Heliconius | erato | hydara |
| SRR4031999 | Colombia | Heliconius | erato | hydara |
| SRR4032000 | Colombia | Heliconius | erato | hydara |
| SRR4032061 | Colombia | Heliconius | erato | hydara |
| SRR4032068 | Colombia | Heliconius | erato | hydara |
| SRR4031995 | Panama | Heliconius | erato | demophoon |
| SRR4031997 | Panama | Heliconius | erato | demophoon |
| SRR4032001 | Panama | Heliconius | erato | demophoon |
| SRR4032002 | Panama | Heliconius | erato | demophoon |
| SRR4032093 | Panama | Heliconius | erato | demophoon |
| SRR4032044 | Ecuador | Heliconius | erato | lativitta |
| SRR4032045 | Ecuador | Heliconius | erato | lativitta |
| SRR4032046 | Ecuador | Heliconius | erato | lativitta |
| SRR4032047 | Ecuador | Heliconius | erato | lativitta |
| SRR4032053 | Ecuador | Heliconius | erato | lativitta |
| SRR4032032 | Peru | Heliconius | erato | favorinus |
| SRR4032056 | Peru | Heliconius | erato | favorinus |
| SRR4032057 | Peru | Heliconius | erato | favorinus |
| SRR4032058 | Peru | Heliconius | erato | favorinus |
| SRR4032059 | Peru | Heliconius | erato | favorinus |
| SRR4032003 | Brazil | Heliconius | erato | phyllis |
| SRR4032004 | Brazil | Heliconius | erato | phyllis |
| SRR4032006 | Brazil | Heliconius | erato | phyllis |
| SRR4032007 | Brazil | Heliconius | erato | phyllis |
| ERR2298201 | French Guiana | Heliconius | melpomene | melpomene |
| ERR2298204 | French Guiana | Heliconius | melpomene | melpomene |
| ERR2298207 | French Guiana | Heliconius | melpomene | melpomene |
| ERR2298214 | French Guiana | Heliconius | melpomene | melpomene |
| ERR2298216 | French Guiana | Heliconius | melpomene | melpomene |
| ERR2298235 | Colombia | Heliconius | timareta | florencia |
| ERR2298233 | Colombia | Heliconius | timareta | florencia |
| ERR2298234 | Colombia | Heliconius | timareta | florencia |
| ERR2298237 | Colombia | Heliconius | timareta | florencia |
| ERR2298236 | Colombia | Heliconius | timareta | florencia |
