# Supplementary material for: Cortex cis-regulatory switches establish scale colour identity and pattern diversity in Heliconius
Source: eLife. 2021 Jul 19;10:e68549. doi: 10.7554/eLife.68549 (PMC8289415; doi:10.7554/eLife.68549)
Supplement: Figure 8—source data 2. — The BovB-like TE element is indicated in blue; The Helitron-like fragment in orange. Both are absent from the H. melpomene melpomene sequence. [file elife-68549-fig8-data2.docx]

Consensus sequences recovered from Sanger sequencing across the *H.* *melpomene/timareta* CRE. The BovB-like TE element is indicated in blue; The Helitron-like fragment in orange. Both are absent from the *H. melpomene melpomene* sequence.

>*H. melpomene rosina* CRE

ACTTCCAACATTCCGCAACATTTCATATTTGGATAGACCACCTTCGCTAATCAGTTATCAGTAATTAAAATGTACATAATTGTATGAACAAACAGCTTACAATGTAAGGAGTGGAAAACAATATAAGTAAAACAATATAAGTAAAGTTTCCTCGTATAAATTCATATAACGTTACGTTCAGGTTATTATTTATTTTAAGTACAAATGATGATAAGACTAATGATATGCTGTAATATGTTGCATATAGGGTATCTTCCTCCTGGCATTAATCCCGGCTATTGCCAGGGTCTGCCCTCCTACTCAACCTACTCCACTTTGCACGGTCTTTTGCGTCCTCGGTGGTTAGACCATTGGCTCTCATGTCCTGCATCACGACATCCAGCCAGCGCTTCCTTGGGCGTCGAGGAGGTCTCTCTCCAGGAACTGATATAGAAAGGCACTTGCATCCGACATAGTTCTTAGGCCGGCGCGTACCATGGCCAAACCATTTCAGACGACGCTCTTGGAGCTTATCACGGACACGGACGGACCCCAAGACTACCTCGAATGAATGTGTTGCGTATGCGGTCAGACCGCGTTACGCCGCACATCCACCTCAGCATCTTTATTTCCGTGACGTGAAGCTCCTGAGTGTGCCGAGATAGTGCCGGCCAACATTCGCTGCCGTATAAAAGAACCGGTCGGATGATGCTCTTGTATATCAGCCCCTTGAGCTTGGGCGGTATTCTGCGGTCGCAGACCACACCAGTGACCTCCCGCCATTTGGCCCAGGCAGCGCTTATCCGGCCTTGGACATCGTGATCGATGCCTCCAGACTCGTGCATAACGGTTCCAAGGTACCTGAACTTTTCCGACTTAACGGCTGGCTCAGGACCTATAAGGATCGTGCTCGAGTCCGGGCTCCCGCAGGCTATGGCCATGGCCATGTTGCATATAGGGTATATTTTTCAATACTAAGGATTTTGGTGGTCTATCAATTAAAATAAAATTTTCTATGTTAATTATCTTACTGTTATATTTTTTCGATTTTATACCTAACTAGCGACCCTCTTGCGGCTTCGCCCGCTTTTACTACTGGATTATTCATATAATGTATGCTTGCAAAGCACTTAAGATAATGTGAAAATTATTTAAACCCTAATGCAACCCGCATTTTCGTAGTTACTGCTACTTCATTATATTCATTTTTTAACTTTTAATGAACGTTATCATTAAGTCTTATGGCAATTTTGTATTGAAACAAAACTGATCGTAATTTTTTCTAAAAAAAAATACACAGAAATCATTTTATTAAAAAGATATAAAGCCAAATAATAAATAAAAGTTGTATAATACAGTATAACGTACATAGAAAATTTAAATAACCTGAGTTACACCACTTTGCCTAGACGCCCCGCACTGGGTGGCTGACGTCAATT

>*H. melpomene amaryllis* CRE

ACTTCGCTATCAGTTATCAGTAATTAAAATGTACATAATTGTATGAACAAACAGCTTACAATGTAAGGAGTGGAAAACAATATAAGTAAAACAATATAAGTAAAGTTTCCTCGTATAAATTCATATAACGTTACGTTCAGGTTATTATTTATTTTAAGTACAAATGATGATAAGACTAATGATATGCTGTAATATGTTGCATATAGGGTATCTTCCTCCTGGCATTAATCCCGGCTATTGCCAGGGTCTGCCCTCCTACTCAACCTACTCCACTTTGCACGGTCTTTTGCGTCCTCGGTGGTTAGACCATTGGCTCTCATGTCCTGCATCACGACATCCAGCCAGCGCTTCCTTGGGCGTCGAGGAGGTCTCTCTCCAGGAACTGATATAGAAAGGCACTTGCATCCGACATAGTTCTTAGGCCGGCGCGTACCATGGCCAAACCATTTCAGACGACGCTCTTGGAGCTTATCCGCTACGTCACGGACCCCAAGACTACCTCGAATGAATGTGTTGCGTATGCGGTCAGACCGCGTTACGCCGCMCATCCACCTCAGCATCTTTATTTCCGTGACGTGAAGCTCCTGAGTGTGCCGAGATAGTGCCGGCCAACATTCGCTGCCGTATAAAAGAACCGGTCGGATRATGCTCTTGTATATCAGCCCCTTGAGCTTGGGCGGTATTCTGCGGTCGCAGACCACACCAGTGACCTCCCGCCATTTGGCCCAGGCAGCGCTTATCCGGCCTTGGACATCGTGATCGATGCCTCCAGACTCGTGCATAACGGTTCCAAGGTACCTGAACTTTTCCGACTTAACGGCTGGCTCAGGACCTATAAGGATCGTGCTCGAGTCCGGGCTCCCGCAGGCCATGGCCATGGCCATGTTGCATATAGGGTATATTTTTCAATACTAAGGATTTTGGTGGTCTATCGATTAAAATAAAATTTTCTATGTTAATTATCTTACTGTTATATTTTTTCGATTTTATACCTAACTAGCGACCCTCTTGCGGCTTCGCCCGCTTTTACTACTGGATTATTTATATAATGTATGCTTGCAAAGCATTTAACATAATGTGAAAATTATTTAAACCCTAATGCAACCCGCATTTTCGTAGTTACTGCTACTTCATTATATTCATTTTTTAACTTTTAATGAACGTTATCATTAAGTCTTATGGWAATTTTGTATTGAAACAAAACTGATCGTAATTTTTTCTAAAAAAAAATACACAGAAATCATTTTATTAAAAAGATATAAAGCCAAATAATAAATAAAAGTTGTATAATACAGTATAACGTACATAGAAAATTTAAATAACCTGAGTACACCA

>*H. melpomene bellula* CRE

CACTTCGCTATCAGTTATCAGTAATTAAAATGTACATAATTGTATGAACAAACAGCTTACAATGTAAGGAGTGGAAAACAATATAAGTAAAACAATATAAGTAAAGTTTCCTCGTATAAATTCATATAACGTTACGTTCAGGTTATTATTTATTTTAAGTACAAATGATGATAAGACTAATGATATGCTGTAATATGTTGCATATAGGGTATCTTCCTCCTGGCATTAATCCCGGCTATTGCCAGGGTCTGCCCTCCTACTCAACCTACTCCACTTTGCACGGTCTTTTGCGTCCTCGGTGGTTAGACCATTGGCTCTCATGTCCTGCATCACGACATCCAGCCAGCGCTTCCTTGGGCGTCGAGGAGGTCTCTCTCCAGGAACTGATATAGAAAGGCACTTGCATCCGACATAGTTCTTAGGCCGGCGCGTACCATGGCCAAACCATTTCAGACGACGCTCTTGGAGCTTATCCGCTACGTCACGGACCCCAAGACTACCTCGAATGAATGTGTTGCGTATGCGGTCAGACCGCGTTACGCCGCCCATCCACCTCAGCATCTTTATTTCCGTGACGTGAAGCTCCTGAGTGTGCCGAGATAGTGCCGGCCAACATTCGCTGCCGTATAAAAGAACCGGTCGGATAATGCTCTTGTATATCAGCCCCTTGAGCTTGGGCGGTATTCTGCGGTCGCAGACCACACCAGTGACCTCCCGCCATTTGGCCCAGGCAGCGCTTATCCGGCCTTGGACATCGTGATCGATGCCTCCAGACTCGTGCATAACGGTTCCAAGGTACCTGAACTTTTCCGACTTAACGGCTGGCTCAGGACCTATAAGGATCGTGCTCGAGTCCGGGCTCCCGCAGGCCATGGCCATGGCCATGTTGCATATAGGGTATATTTTTCAATACTAAGGATTTTGGTGGTCTATCGATTAAAATAAAATTTTCTATGTTAATTATCTTACTGTTATATTTTTTCGATTTTATACCTAACTAGCGACCCTCTTGCGGCTTCGCCCGCTTTTACTACTGGATTATTTATATAATGTATGCTTGCAAAGCATTTAACATAATGTGAAAATTATTTAAACCCTAATGCAACCCGCATTTTCGTAGTTACTGCTACTTCATTATATTCATTTTTTAACTTTTAATGAACGTTATCATTAAGTCTTATGGTAATTTTGTATTGAAACAAAACTGATCGTAATTTTTTCTAAAAAAAAATACACAGAAATCATTTTATTAAAAAGATATAAAGCCAAATAATAAATAAAAGTTGTATAATACAGTATAACGTACATAGAAAATTTAAATAACCTGAGTACACCA

>*H. timareta tristero* CRE

TTCGCTATCAGTTATCAGTAATTAAAATGTACATAATTGTATGAACAAACAGCTTACAATGTAAGGAGTGGAAAACAATATAAGTAAAACAATATAAGTAAAGTTTMCTCGTATAAATTCATATAACGTTACGTTCAGGTTATTATTTATTTTAAGTACAAATGATGATAAGACTAATGATATGCTGTAAT

ATGTTGCATATAGGGTATCTTCCTCCTGGCATTAATCCCGGCTATTGCCAGGGTCTGCCCTCCTACTCAACCTACTCCACTTTGCACGGTCTTTTGCGTCCTCGGTGGTTAGACCATTGGCTCTCATGTCCTGCATCACGACATCCAGCCAGCGCTTCCTTGGGCGTCGAGGAGGTCTCTCTCCAGGAACTGATATAGAAAGGCACTTGCATCCGACATAGTTCTTAGGCCGGCGCGTACCATGGCCAAACCATTTCAGACGACGCTCTTGGAGCTTATCCGCTACGTCACGGACCCCAAGACTACCTCGAATGAATGTGTTGCGTATGCGGTCAGACCGCGTTACGCCGCACATCCACCTCAGCATCTTTATTTSMGTGACGTGAAGCTCCTGAGTGTGCCGAGATAGTGCCGGCCAACATTCGCTGCCGTATAAAAGAACCGGTCGGATGATGCTCTTGTATATCAGCCCCTTGAGCTTGGGCGGTATTCTGCGGTCGCAGACCACACCRGTGACCTCCCGCCATTTGGCCCAGGCAGCGCTTATCTGGCCTTGGACATCGTGATCGATGCCTCCAGACTCGTGCATAACGGTTCCAAGGTACCTGAACTTTTCCGACTTAACGGCTGGCTCAGGACCTATAAGGATCGTGCTCGAGTCCGGGCTCCCGCAGGYYATGGCCATGGCCATGTTGCATATAGGGTATATTTTTCAATACTAAGGATTTTGGTGGTCTATCARTTAAAATAAAATGTTCTATGTTAATTATCTTACTGYTATATTTTTTCGAYYYTATACCTADCTAGCGAYCCTCWTGCRRCTTCGSCYGNCTAAGCACTTAAGATAATGTGAAAATTATTTAAACCCTAATGCAACCCGCATTTTYGTAGTTACTGCTACTTCATTATATTCATTTTTTAACTTTTAATGAACGTTATCATTAAGTCTTATGGMAATTTTGTATTGAAACAAAACTGATCGTAATTTTTTYTAAAAAAAAATACACAGAAATCATTTTATTAAAAAGATATAAAGCCAAATAATAAATAAAAGTTGTATAATACAGTATAACGTACATAGAAAATTTAAATAACCTGAGTAC

>*H. melpomene melpomene* CRE

ACTTCCAACATTCCGCAACATTTCTTATTTGGTTAGACATTAGACCAACATCGCTAATCATTTATCAATAATTAAAATGTACATAATTGTATGAACAAACAGCTTACAATATAAGGATGGAACTAAATAAAGTTTTATTATTATTATTTTTTATTTATGGAAAACAATTCATACATTATTAAGAGTAATAAATAAGTAAAGTTTCCTCGTATAAATTCATATAACGTTACGTTCAAGTTATTATTTATTTTAAGTACAAATGATGATAACACTAAAAATATGCTGTAATATGTTGCATATAGGGTATATTTTTCAATACTAAGGAAAATGGTGGTCTATCAATTAAAATAAAATTTTCTATGTTAATTATCTTACACACCTTACATCTAGTTATATTTTTTCGATTGTATACCTAATCAACCATTAATTAGTATTCAAAAGTATGATAATTTCTACATAACGCGAAAAAAGGTACCGTTTTCAAGAAGCATGTGTTCTAGTCCTTTTCTTTCCTTAGAGTATGAATAATACATTAAATAAAGGTGGCATGGCATGGTTCATGGCTGCTACTTCATTATATTCATTTATTAACTTTTATGACGTTCATTAAGTCTTATGGCAATTTTGTATTGAAACAAAACTTATTGTAATTTTTTCTAAAAAAAATACACAGGAATCGTTTTATTAAAAAGATATAAAGCCAAATAATAAATAAAACTTGTATAATACAGTATAACGTACGTGGAAAATTTAAATAACCTGAGTTACACCACTTTGCTTGGACGCCCCGCACTGGGTGGCTGACGTCAATT
