# Supplementary material for: Cortex cis-regulatory switches establish scale colour identity and pattern diversity in Heliconius
Source: eLife. 2021 Jul 19;10:e68549. doi: 10.7554/eLife.68549 (PMC8289415; doi:10.7554/eLife.68549)
Supplement: Figure 9—source data 1. [file elife-68549-fig9-data1.docx]

**MELPOMENE SCALE WIDTH**

|  | **cythera, black WT** | **cythera, white WT** | **cythera, white Mutant** | **rosina, black WT** | **rosina, yellow mutant** |
| --- | --- | --- | --- | --- | --- |
| **cythera, white WT** | 0.25579 | - | - | - | - |
| **cythera, white Mutant** | 0.40818 | 0.60473 | - | - | - |
| **rosina, black WT** | 0.00063 | 0.03835 | 0.00047 | - | - |
| **rosina, yellow mutant** | 0.00936 | 0.40317 | 0.03835 | 0.00047 | - |
| **rosina, yellow WT** | 0.00067 | 0.07611 | 0.01998 | 0.10855 | 0.25579 |

**MELPOMENE WIDTH MELPOMENE SCALE LENGTH**

|  | **cythera, black WT** | **cythera, white WT** | **cythera, white Mutant** | **rosina, black WT** | **rosina, yellow mutant** |
| --- | --- | --- | --- | --- | --- |
| **cythera, white WT** | 0.00253 | - | - | - | - |
| **cythera, white Mutant** | 0.419 | 0.04351 | - | - | - |
| **rosina, black WT** | 1.90E-06 | 3.00E-06 | 0.00012 | - | - |
| **rosina, yellow mutant** | 5.50E-08 | 7.80E-08 | 2.10E-05 | 0.01305 | - |
| **rosina, yellow WT** | 3.40E-05 | 9.30E-06 | 0.0003 | 0.02436 | 0.91489 |
| **MELPOMENE SERRATIONS** | | | | | |
|  | **cythera, black WT** | **cythera, white WT** | **cythera, white Mutant** | **rosina, black WT** | **rosina, yellow mutant** |
| **cythera, white WT** | 0.4766 | - | - | - | - |
| **cythera, white Mutant** | 1 | 0.4489 | - | - | - |
| **rosina, black WT** | 0.1147 | 0.674 | 0.0575 | - | - |
| **rosina, yellow mutant** | 0.6758 | 0.6758 | 0.5145 | 0.4164 | - |
| **rosina, yellow WT** | 0.0084 | 0.0815 | 0.0036 | 0.0867 | 0.0120 |
| **MELPOMENE RIDGE PERIODICITY** | | | | | |
|  | **cythera, black WT** | **cythera, white WT** | **cythera, white Mutant** | **rosina, black WT** | **rosina, yellow mutant** |
| **cythera, white WT** | 0.05492 | - | - | - | - |
| **cythera, white Mutant** | 0.92072 | 0.11904 | - | - | - |
| **rosina, black WT** | 0.18957 | 0.01475 | 0.78471 | - | - |
| **rosina, yellow mutant** | 0.00819 | 0.00175 | 0.11904 | 0.044 | - |
| **rosina, yellow WT** | 0.00018 | 0.00029 | 0.0056 | 0.00018 | 0.01524 |

**MELPOMENE CROSSRIB PERIODICITY**

**cythera, black WT**

**rosina, black WT** 7.1e-05

**MELPOMENE MICRORIB PERIODICITY**

|  | **cythera, white WT** | **cythera, white Mutant** | **rosina, yellow mutant** |
| --- | --- | --- | --- |
| **cythera, white Mutant** | 0.083 | - | - |
| **rosina, yellow mutant** | 0.667 | 0.083 | - |
| **rosina, yellow WT** | 0.083 | 0.686 | 0.083 |
