# Supplementary material for: Cortex cis-regulatory switches establish scale colour identity and pattern diversity in Heliconius
Source: eLife. 2021 Jul 19;10:e68549. doi: 10.7554/eLife.68549 (PMC8289415; doi:10.7554/eLife.68549)
Supplement: Figure 9—source data 2. [file elife-68549-fig9-data2.docx]

| **ERATO SCALE WIDTH** | | | | | | |
| --- | --- | --- | --- | --- | --- | --- |
|  | **cyrbia, black** | **cyrbia, white** | **cyrbia, white** | **demophoon, black** | **demophoon, yellow** | **demophoon, yellow** |
|  | **WT** | **WT** | **Mutant** | **WT** | **Mutant** | **WT** |
| **cyrbia, white WT** | 6.00E-05 | - | - | - | - | - |
| **cyrbia, white** | 0.52849 | 0.00057 | - | - | - | - |
| **Mutant** |  |  |  |  |  |  |
| **demophoon, black** | 0.51944 | 0.00015 | 0.37403 | - | - | - |
| **WT** |  |  |  |  |  |  |
| **demophoon, yellow** | 0.60363 | 6.70E-05 | 0.50041 | 0.51944 | - | - |
| **Mutant** |  |  |  |  |  |  |
| **demophoon, yellow** | 0.27121 | 2.00E-06 | 0.86485 | 0.14757 | 0.09625 | - |
| **WT** |  |  |  |  |  |  |
| **hydara, red WT** | 0.01967 | 0.00057 | 0.85909 | 0.0102 | 0.04998 | 0.85909 |
| **ERATO SCALE LENGTH** | | | | | | |
|  | **cyrbia, black** | **cyrbia, white** | **cyrbia, white** | **demophoon, black** | **demophoon, yellow** | **demophoon, yellow** |
|  | **WT** | **WT** | **Mutant** | **WT** | **Mutant** | **WT** |
| **cyrbia, white WT** | 0.3587 | - | - | - | - | - |
| **cyrbia, white Mutant** | 0.4991 | 0.8479 | - | - | - | - |
| **demophoon, black** | 0.0014 | 0.4521 | 0.3587 | - | - | - |
| **WT** |  |  |  |  |  |  |
| **demophoon, yellow** | 0.4958 | 0.0961 | 0.1443 | 0.0002 | - | - |
| **Mutant** |  |  |  |  |  |  |
| **demophoon, yellow** | 0.093 | 0.0206 | 0.1021 | 1.50E-05 | 0.4397 | - |
| **WT** |  |  |  |  |  |  |
| **hydara, red WT** | 0.1443 | 0.8479 | 1 | 0.1306 | 0.075 | 0.0176 |
| **ERATO PRONG NUMBER** | | | | | | |
|  | **cyrbia, black** | **cyrbia, white** | **cyrbia, white** | **demophoon, black** | **demophoon, yellow** | **demophoon, yellow** |
|  | **WT** | **WT** | **Mutant** | **WT** | **Mutant** | **WT** |
| **cyrbia, white WT** | 0.00028 | - | - | - | - | - |
| **cyrbia, white** | 0.15704 | 0.00241 | - | - | - | - |
| **Mutant** |  |  |  |  |  |  |
| **demophoon, black** | 0.00151 | 0.00014 | 0.04219 | - | - | - |
| **WT** |  |  |  |  |  |  |
| **demophoon, yellow** | 0.03611 | 6.00E-05 | 0.12531 | 0.06224 | - | - |
| **Mutant** |  |  |  |  |  |  |
| **demophoon, yellow** | 0.06115 | 6.00E-05 | 0.80401 | 0.00655 | 0.06115 | - |
| **WT** |  |  |  |  |  |  |
| **hydara, red WT** | 0.01286 | 0.00241 | 0.58257 | 0.06224 | 0.19112 | 0.69988 |
| **ERATO RIDGE PERIODICITY** | | | | | | |
|  | **cyrbia, black** | **cyrbia, white** | **cyrbia, white** | **demophoon, black** | **demophoon, yellow** | **demophoon, yellow** |
|  | **WT** | **WT** | **Mutant** | **WT** | **Mutant** | **WT** |
| **cyrbia, white WT** | 0.02624 | - | - | - | - | - |
| **cyrbia, white** | 0.00728 | 0.05495 | - | - | - | - |
| **Mutant** |  |  |  |  |  |  |
| **demophoon, black** | 0.3655 | 0.01305 | 0.00036 | - | - | - |
| **WT** |  |  |  |  |  |  |
| **demophoon, yellow** | 0.00189 | 0.01142 | 0.50312 | 0.00094 | - | - |
| **Mutant** |  |  |  |  |  |  |
| **demophoon, yellow** | 0.25182 | 0.00582 | 0.00021 | 0.06393 | 0.00021 | - |
| **WT** |  |  |  |  |  |  |
| **hydara, red WT** | 0.02008 | 0.05495 | 0.05495 | 0.06247 | 0.10949 | 0.00617 |
| **ERATO CROSSRIB PERIODICITY** | |  | |  | | |
|  | | **cyrbia, black WT** | | **demophoon, black WT** | | |
| **demophoon, black WT** | | 0.00042 | | - | | |
| **hydara, red WT** | | 0.00163 | | 0.00015 | | |
| **ERATO MICRORIB PERIODICITY** | |  |  | |  | |
|  | | **wt_white.cyr** | **mut_white.cyr** | | **mut_yellow.dem** | |
| **mut_white.cyr** | | 0.194 | - | | - | |
| **mut_yellow.dem** | | 0.398 | 0.801 | | - | |
| **wt_yellow.dem** | | 0.013 | 0.194 | | 0.27 | |
