# Supplementary material for: Cortex cis-regulatory switches establish scale colour identity and pattern diversity in Heliconius
Source: eLife. 2021 Jul 19;10:e68549. doi: 10.7554/eLife.68549 (PMC8289415; doi:10.7554/eLife.68549)
Supplement: Transparent reporting form [file elife-68549-transrepform.docx]

***eLife’s* transparent reporting form**

We encourage authors to provide detailed information *within their submission* to facilitate the interpretation and replication of experiments. Authors can upload supporting documentation to indicate the use of appropriate reporting guidelines for health-related research (see [EQUATOR Network](http://www.equator-network.org/%20)), life science research (see the [BioSharing Information Resource](https://biosharing.org/)), or the [ARRIVE guidelines](http://www.plosbiology.org/article/info:doi/10.1371/journal.pbio.1000412) for reporting work involving animal research. Where applicable, authors should refer to any relevant reporting standards documents in this form.

If you have any questions, please consult our Journal Policies and/or contact us: [editorial@elifesciences.org](mailto:editorial@elifesciences.org).

**Sample-size estimation**

- You should state whether an appropriate sample size was computed when the study was being designed
- You should state the statistical method of sample size computation and any required assumptions
- If no explicit power analysis was used, you should describe how you decided what sample (replicate) size (number) to use

No statistical analyses requiring sample size computation were performed in this study. Sample sizes were chosen based on standard practices for each type of dataset.

Please outline where this information can be found within the submission (e.g., sections or figure legends), or explain why this information doesn’t apply to your submission:

**Replicates**

- You should report how often each experiment was performed
- You should include a definition of biological versus technical replication
- The data obtained should be provided and sufficient information should be provided to indicate the number of independent biological and/or technical replicates
- If you encountered any outliers, you should describe how these were handled
- Criteria for exclusion/inclusion of data should be clearly stated
- High-throughput sequence data should be uploaded before submission, with a private link for reviewers provided (these are available from both GEO and ArrayExpress)

Please outline where this information can be found within the submission (e.g., sections or figure legends), or explain why this information doesn’t apply to your submission:

Sample size for RNA-seq analysis was performed under the standard assumption of sufficiency with three biological replicates per tissue. Details on RNA-Seq analysis can be found in the methods under the “Tissue sampling and RNA-seq” section. The RNA-Seq datasets generated and/or analysed during the current study are available in the SRA repository (PRJNA552081).

Similarly, for the ATAC-Seq analyses, we generated three biological replicate libraries per butterfly morph/species. This is standard practice for these types of data. In order to plot the traces to assess individual peaks, we averaged the read count across replicates. See “ATAC-Seq” section under Methods for further details. Each library is available under the accession numbers provided in supplementary File 12 – Table S12.

Measurements on scales were performed on at least 10 individual scales of each type. See “Morphometrics analysis” under the method section for details. The R script used for the analysis is available at <https://github.com/Hanliconius?tab=repositories>, under “nanomorphometrics”.

For CRISPR phenotypes we generated at least three biological replicates per species, with a total of 43 mutant individuals recovered across all species/motphs. Details of injections can be found in the supplementary File 8 and resulting mutants in supplementary file 9.

**Statistical reporting**

- Statistical analysis methods should be described and justified
- Raw data should be presented in figures whenever informative to do so (typically when N per group is less than 10)
- For each experiment, you should identify the statistical tests used, exact values of N, definitions of center, methods of multiple test correction, and dispersion and precision measures (e.g., mean, median, SD, SEM, confidence intervals; and, for the major substantive results, a measure of effect size (e.g., Pearson's r, Cohen's d)
- Report exact p-values wherever possible alongside the summary statistics and 95% confidence intervals. These should be reported for all key questions and not only when the p-value is less than 0.05.

Please outline where this information can be found within the submission (e.g., sections or figure legends), or explain why this information doesn’t apply to your submission:

Statistical significance for RNA-seq data was computed using the standard approach of applying a generalized linear model with the R package DESeq2. DESeq2 fits negative binomial generalized linear models for each gene and uses the Wald test for significance testing. In total, we sequenced 18 samples representing three developmental stages (larval, 36h +/-1.5h (Day 1 pupae) and 60h +/- 1.5h (Day 2 pupae)) from two morphs in each of the two species, with hindwings divided into two parts for the pupal stages. Further details of the samples are provided in the manuscript in the first section of the results (starting at line 119) and methodology sections under (Tissue sampling and RNA-seq). Significant differences are reported in Figure 2 of the manuscript

indicated with a * when the Wald test adjusted p=<0.05.

For the non-parametric Wilcoxon test applied to scale measurements presented

in Supplementary File 15 - Figure S15, significance for each pairwise comparison is reported in Table S15. A non-parametric test was used as the data analysed was not normally distributed (we tested for normality using a Shapiro-Wilk test). Individual measurements are provided in an associated file on the Dryad repository as well as p- values for each test conducted, and data spread and number indicated by violin plots.

(For large datasets, or papers with a very large number of statistical tests, you may upload a single table file with tests, Ns, etc., with reference to sections in the manuscript.)

**Group allocation**

- Indicate how samples were allocated into experimental groups (in the case of clinical studies, please specify allocation to treatment method); if randomization was used, please also state if restricted randomization was applied
- Indicate if masking was used during group allocation, data collection and/or data analysis

Please outline where this information can be found within the submission (e.g., sections or figure legends), or explain why this information doesn’t apply to your submission:

Sequencing samples were allocated on the basis of wing phenotype for each species where RNA/nuclei were extracted in replicates and sequenced per wing section and, in caterpillars, on the basis of the presence/absence of a yellow hindwing bar.

Scales for SEM analysis were allocated on the basis of wing position, and phenotype status (black, yellow) and whether they were wild-type or mutant scales.

**Additional data files (“source data”)**

- We encourage you to upload relevant additional data files, such as numerical data that are represented as a graph in a figure, or as a summary table
- Where provided, these should be in the most useful format, and they can be uploaded as “Source data” files linked to a main figure or table
- Include model definition files including the full list of parameters used
- Include code used for data analysis (e.g., R, MatLab)
- Avoid stating that data files are “available upon request”

Please indicate the figures or tables for which source data files have been provided:

All relevant source data and R scripts are available on the associated Dryad repository. https://datadryad.org/stash/share/TWYFX0wh9Q3LvlMClbGrLhicsKXFeMgFnm4ddj5L8Ss
